# Comprehensiveness and validity of a multidimensional assessment in patients with chronic low back pain: a prospective cohort study

**DOI:** 10.1186/s12891-021-04130-x

**Published:** 2021-03-20

**Authors:** Thomas Benz, Susanne Lehmann, Achim Elfering, Peter S. Sandor, Felix Angst

**Affiliations:** 1 Research Department, Rehaklinik Bad Zurzach, Zurzach Care Group, Quellenstrasse 34, Bad Zurzach, Switzerland; 2 Institute of Psychology, University of Bern, Fabrikstrasse 8, Bern, Switzerland; 3 Graduate School for Health Sciences, University of Bern, Bern, Switzerland; 4 Institute of Physiotherapy, School of Health Professions, Zurich University of Applied Sciences, Winterthur, Switzerland

**Keywords:** Multidimensional assessment, Validity, Chronic low back pain, Measurement scales, Patient-reported outcome measurements, Performance-based outcome measurements

## Abstract

**Background:**

Chronic low back pain is a multidimensional syndrome affecting physical activity and function, health-related quality of life and employment status. The aim of the study was to quantify the cross-sectional and longitudinal validity of single measurement scales in specific construct domains and to examine how they combine to build a comprehensive outcome, covering the complex construct of chronic low back pain before and after a standardized interdisciplinary pain program.

**Methods:**

This prospective cohort study assessed 177 patients using the Short Form 36 (SF-36), the Multidimensional Pain Inventory (MPI), the Symptom Checklist-90-Revised (SCL-90-R), the Oswestry Disability Index (ODI), and 2 functional performance tests, the Back Performance Scale (BPS) and the 6-Minute Walking Distance (6MWD). The comprehensiveness and overlap of the constructs used were quantified cross-sectionally and longitudinally by bivariate correlations, exploratory factor analysis, and effect sizes.

**Results:**

The mean age of the participants was 48.0 years (+/− 12.7); 59.3% were female. Correlations of baseline scores ranged from r = − 0.01 (BPS with MPI Life control) to r = 0.76 (SF-36 Mental health with MPI Negative mood). SF-36 Physical functioning correlated highest with the functional performance tests (r = 0.58 BPS, 0.67 6MWD) and ODI (0.56). Correlations of change scores (difference of follow-up – baseline score) were consistent but weaker. Factor analysis revealed 2 factors: “psychosocial” and “pain & function” (totally explained variance 44.0–60.9%). Psychosocial factors loaded strongest (up to 0.89 SCL-90-R) on the first factor, covering 2/3 of the explained variance. Pain and function (ing) loaded more strongly on the second factor (up to 0.81 SF-36 Physical functioning at follow-up). All scales showed improvements, with effect sizes ranging from 0.16–0.67.

**Conclusions:**

Our results confirm previous findings that the chronic low back pain syndrome is highly multifactorial and comprises many more dimensions of health and quality of life than merely back-related functioning. A comprehensive outcome measurement should include the predominant psychosocial domain and a broad spectrum of measurement constructs in order to assess the full complexity of the chronic low back syndrome. Convergence and divergence of the scales capture the overlapping contents and nuances within the constructs.

## Background

Low back pain is the leading cause of disability worldwide; it is associated with disability including impairments (e.g. loss of function), activity limitations, and participation, e.g. in social activities and employment [1]. Between 1990 and 2013, low back pain was globally the top cause of years lived with disability [1]. Although the prognosis of unspecific low back pain is good, for some patients the pain becomes persistent, reducing their health-related quality of life (HRQoL), including their physical, mental, emotional and social functioning [2, 3]. For persistent, chronic low back pain (CLBP), comprehensive treatment integrating biopsychosocial features is recommended [4–6]. Therefore, not only disease-specific but also the comprehensive measurement of pain and pain-related psychosocial co-factors is needed.

The consensus statement of the VAPAIN (Validation and Application of a patient-relevant core set of outcome domains to assess multimodal PAIN therapy) expert panel, which specifically addressed the interdisciplinary multimodal pain treatment of chronic pain, recommended that the measurement of psychosocial factors should not be confined to anxiety and depression but include further distressing emotions, and social participation [7]. Other consensus statements advocate the combined use of patient-reported outcome measurements (PROMs) and performance-based measures (PBMs) to obtain complementary information [8, 9]. However, a systematic review of the psychometric measurement properties of instruments used to measure HRQoL in CLBP found that evidence regarding PROMs and the instruments’ validity was largely missing [10].

Validity is an instrument’s most important psychometric property: it proves whether the tool measures what it is designed to measure [11]. In CLBP, there is some evidence of fair to good validity when two to three instruments are compared, as is the case in most validity studies in specific measurement domains [12–17]. The combination of more than three instruments to capture the multidimensionality of CLBP has not been tested to date. In addition, we do not know how single validated measurement scales work together and combine to build a valid and comprehensive multidimensional assessment of the complexity of CLBP. An expert panel concluded that “composite outcome measures may move us closer to important outcomes” but more data on their performance are needed in terms of reliability, validity (including longitudinal responsiveness), and prognostic value [18].

The aim of the study was to quantify the cross-sectional and longitudinal validity of single measurement scales in specific construct domains and to examine how they combine to build a comprehensive outcome, covering the complex construct of chronic low back pain before and after a standardized interdisciplinary pain program. The study contributes to the knowledge of construct overlap and of a composite assessment of the complexity of the CLBP syndrome.

## Methods

### Patients

The patients in this study were recruited at the pain center of the rehabilitation clinic “Rehaklinik” in Bad Zurzach, Switzerland. All patients were referred to the Zurzach Interdisciplinary Pain Program (ZISP) by their family physician or rheumatologist. Between November 2010 and May 2019, patients with chronic non-specific low back pain who attended the ZISP for the first time were asked to participate in the study. Further inclusion criteria were age ≥ 18 years and persistent pain for ≥3 months at inclusion in the program. Exclusion criteria were severe somatic or mental conditions that prevented participation in the pain program, insufficient German language skills (reading and writing) for completion of the assessment tool, second participation in the program, and refusal to participate in the pain program or the study.

Before joining the ZISP and on the basis of their admission reports, potential participants had been contacted by telephone to evaluate the inclusion and exclusion criteria. Patients of whom the oral German was insufficient to follow the group program (an exclusion criterion of the ZISP) were admitted to a different, individualized pain program in our clinic.

Written informed consent was obtained from all participants. The study protocol was approved by the Local Ethics Commission (Health Department of Aarau, Switzerland, EK AG 2008/026).

### Intervention

The ZISP is a standardized inpatient pain management program for the treatment of chronic musculoskeletal pain disorders. The program is comprehensive and interdisciplinary, comprising active physiotherapy, aerobic endurance training, Qigong/tai chi exercises, individual psychotherapy, including cognitive behavioral therapy, participation in a pain coping group, information and education sessions on the pathophysiology of pain mechanisms and the management of chronic disabling pain, relaxation therapy, humor therapy, horticultural therapy, nursing care, and regular medical consultations, including pharmacotherapy. The program is group based, lasts 4 weeks, comprises an average of 20 therapy sessions/units per week and a total of over 100 h of therapy per program. Details of the ZISP have been published elsewhere [19, 20].

### Measures

In the choice of the outcome instruments, tested and documented validity was the most important selection criterion. At the time of the initial project in the mid 1990s, evidence of validity was still thin on the ground and further literature, especially on recommended core sets, did not exist. After the first phase of the project, the choice of measures was revised on the basis of the results of the previous studies in our institution [19–22]. In particular, relative weak responsive scales were eliminated [22].

Recommended core outcome domains for clinical trials of patients with chronic pain in general, and the complex biopsychosocial construct of CLBP specifically, were considered if available. In the meantime several recommendations for instrument sets for standardized outcome measurement in CLBP have been published [18, 23, 24]. So far, no international consensus has been reached however. For this study population with CLBP, enrolled in an interdisciplinary pain treatment program, several core outcome sets were applicable, depending on the focus of assessment, namely low back pain [25], interdisciplinary multimodal pain therapy [7], and chronic pain in general [26]. As a result, HRQoL, and physical, emotional, and social functioning were assessed by 4 PROMs and 2 PBMs (described in detail below), in order to achieve a comprehensive, multidimensional and biopsychosocial assessment of CLBP.

Sociodemographic and potentially confounding parameters, such as age, gender, occupation (working capacity), living conditions, sports habits, and formal education, were recorded at admission to the clinic on a standardized form used in many previous studies [20, 21]. Comorbidities were retrieved from the patient’s medical history. The validated German versions of the following 4 PROMs used in our study were applied [27–30].

The Medical Outcomes Study Short Form 36 Health Survey (SF-36) comprehensively measures physical, mental and psychosocial health and various dimensions of quality of life [31]. This instrument contains 36 items categorized in 8 health domains: Bodily pain, Physical functioning, Role physical, General health, Vitality, Social functioning, Role emotional, and Mental health. The SF-36 is a commonly used and widely recommended generic questionnaire for the self-assessment of HRQoL in chronic pain diseases such as CLBP [12, 32, 33]. It has been extensively tested in CLBP for both validity and reliability [12].

The West Haven-Yale Multidimensional Pain Inventory (MPI) assesses pain and psychosocial and behavioral aspects in patients with chronic pain. The MPI is divided into 11 subscales grouped into 3 sections: 1) Pain impact: Pain severity, Interference due to pain, Life control, Affective distress (synonymously described as negative mood), Support, 2) Response by significant others: Negative, Solicitous and Distracting responses, and 3) Activities: Household chores, Outdoor Work, Activities away from home, and Social activities. An additional general activity level score is calculated by 4 separate activity domains [13]. Excellent reliability and validity in low back pain has been reported [17].

The Symptom Checklist 90-revised (SCL-90-R) is a self-report instrument that measures a broad range of symptoms of psychological distress and psychiatric illness and is widely used with patients with chronic low back pain [14, 29, 34–37]. From the total of 9 scales of the SCL-90-R the following 4 were assessed in our study: Somatization (12 items), Anxiety (10 items), Depression (13 items) and Anger-hostility (6 items). The validity of the SCL-90-R is documented by extensive use in psychiatric conditions.

The Oswestry Disability Index (ODI) is a self-administered condition-specific questionnaire for patients with back pain. It assesses pain-related functional disability by means of 10 items, 9 of which deal with activities of daily living (personal care, lifting, walking, sitting, standing, sleeping, sex life, social life, and travelling) and one covers the intensity of pain. The total ODI score ranges from 0 = no disability to 100 = bedbound [38]. It is one of the most commonly used instruments with good measurement properties (reliability, validity and responsiveness) for evaluating physical functioning and spine-related disability in patients with low back pain [12, 39–41].

In addition to the 4 PROMs described above, we used 2 PBMs. The Back Performance Scale (BPS) is a physical performance assessment instrument covering 5 different activities that are often limited in patients with back pain: the Sock test, Pick-up test, Roll-up test, Fingertip-to-floor test and Lift test. Each test is scored separately on a Likert-scale from 0 to 3; the sum of the scores gives a total maximum score of 15 (=major activity limitations). The 5 tests together capture physical limitation better than separate tests [42]. A detailed description of the 5 tests can be found in the literature [15]. The BPS is a reliable and valid outcome measurement tool [15].

The 6 Minute Walking Distance test (6MWD) measures the distance walked in 6 min on a premeasured, 100 m long, flat walking surface with interval markings every 5 m. The greater the distance covered, the better the performance. The 6MWD is an easy functional performance test requiring minimal equipment [16]. It is recommended for the assessment of physical function in chronic pain trials [9].

### Analysis

The patients were assessed on admission to the clinic (baseline measurement before therapy) and again on discharge from the pain program after 4 weeks’ treatment (follow-up measurement). The instrument-specific “missing rules” had to be fulfilled in order to determine the scales. Thus, at least 50% of the items had to be completed for each of the SF-36 scales, and 3/4 (76%) for the SCL-90-R [29, 43]. Since for the other instruments no missing rules were reported in the initial papers describing the original questionnaires, we applied the 2/3 missing rule (completion of 67% of the items required to determine the score) as previously reported [44]. All analyses were performed using the statistical software package IBM SPSS 25.0 for Windows® (SPSS Inc., Chicago, IL, USA).

In this study, all scores, except those of the 6MWD, were converted into scales ranging from 0 to 100. The score 0 indicates maximum limitation, disability, or symptoms whereas the score 100 means no limitation, disability or symptoms. The purpose was to facilitate comparison between the different scoring systems of the assessment tools included in this study. Mean values with standard deviation and, for effect quantification, effect sizes (ESs) according to Kazis [45] and standardized response mean (SRM) according to Liang [46] were calculated. The ES according to Kazis is defined as the score difference (follow-up – baseline) divided by the standard deviation of the baseline score [45]. The SRM is defined as the score difference (follow-up – baseline) divided by the standard deviation of the score differences (follow-up – baseline) [46]. For both the ES and the SRM a positive value of > 0.80 is considered as showing a large, 0.50 – 0.79 a moderate, 0.20–0.49 a small, and 0.00–0.19 a very small improvement. A negative ES or SRM reflects worsening.

For the analysis of the construct validity, bivariate Pearson correlations and factor analyses were calculated [11, 47]. The correlation reflects the strength of the association between two variables. There is no generally applied rule for the classification of correlation coefficients, but a correlation coefficient (r) above 0.75 can be considered an excellent association, 0.50–0.75 moderate to good, 0.25–0.50 fair, and 0.00–0.25 little or no relationship [47].

Factor analysis is a multivariate correlation analysis aimed at reducing the number of dimensions, identifying common constructs and explaining the nature of their interrelations [47, 48]. For the extraction of the number of factors of the factor analysis, “Velicer’s minimum average partial (MAP) test” and “parallel analysis” were used [49]. The factor load reflects the construct convergence of the scale to the major underlying, common dimension of the factor. Large factor loads indicate the representation of the same underlying construct, whereas small factor loads do not [49].

We chose two criteria to determine the sample size: first, a factor analysis should comprise at least 5 cases per variable, i.e. 5*20(scales) = 100 patients, in order to be sufficiently determinate [49]. Second, patient recruitment was continued until the sample reached the size at which small effects (ES according to Kazis ≥0.21) were statistically significant from 0, which means that the 95% confidence interval excluded 0 [50].

## Results

### Patients

**Fig. 1:**
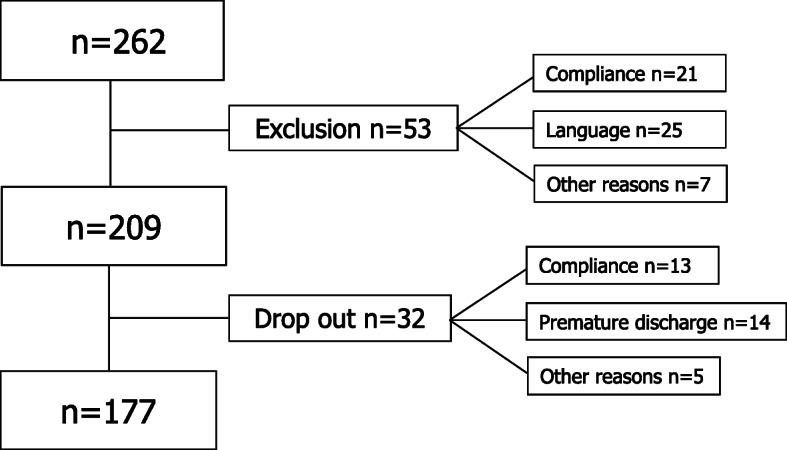
Flow chart of study participants

The sociodemographic variables and disease-relevant characteristics are shown in Table 1. The flow chart of participants is presented in Fig. 1. The complete data of *n* = 177 patients were available. The mean participant was 48.0 years old, female (59.3%), educated to vocational training level (50.3%) and currently not working (46.3%). Typical patients were living with a partner (53.1%), were not regularly involved in sports (46.9%) and suffered from at least one comorbidity (28.8%).

**Table 1:** Socio-demographic and disease-relevant data (*n* = 177)

		n	%
Age	mean +/− sd (years)	177	48.0 (12.7)
Sex	female	105	59.3
Education^a^	Basic school (8–9 years)	52	29.4
	Vocational training	89	50.3
	College/high school/university	33	18.6
Working capacity^b^	Not working	82	46.3
	Part time	49	27.7
	Full time (42 h/week)	37	20.9
Living conditions^c^	Alone	49	27.7
	With partner	94	53.1
	other	32	18.1
Sports, hours/week^d^	None	83	46.9
	< 1	23	13.0
	1–2	35	19.8
	> 2	30	16.9
Comorbidities^e^	none	18	10.2
	1	51	28.8
	2	35	19.8
	3	29	16.4
	4	23	13.0
	≥5	20	11.3

Legend: sd = standard deviation

^a^Missings: *n* = 3

^b^Missings: *n* = 9

^c^Missings: *n* = 2

^d^Missings: *n* = 6

^e^Missings: *n* = 1

### Outcome and comparison of score changes (baseline to follow-up)

At baseline, the highest scores (=best health) on all PROMs were measured on the 4 scales of the SCL-90-R, with the highest mean score on the Anger-hostility dimension (m = 78.5, sd = 17.9) (Table 2). The scores on both the ODI (m = 55.5, sd = 12.4) and the MPI Life control (m = 51.4, sd = 21.0) scales were above 50, whereas all other scores were below 50. The lowest score was on SF-36 Bodily pain with m = 19.0 (sd = 13.9), indicating intense pain. On the BPS the mean score was 51.7 (sd = 22.6) and the 6MWD measured a mean distance of 403.6 m (sd = 155.2).

**Table 2:** Baseline and follow-up scores, score differences, effect sizes and standardized response mean of all assessments

	Baseline (mean)	SD	Follow-up (mean)	SD	Difference (mean)	SD	ES	SRM
**SF-36**								
**Physical functioning (PF)**	47.1	18.6	53.3	20.0	6.2	15.8	0.33	0.39
**Role physical (RP)**	26.3	17.9	36.2	19.6	9.9	20.6	0.55	0.48
**Bodily pain (BP)**	19.0	13.9	29.2	15.0	10.2	13.9	0.73	0.73
**General health (GH)**	42.1	16.4	46.1	18.2	3.9	16.1	0.24	0.25
**Vitality (VT)**	30.4	18.6	42.9	20.3	12.5	18.5	0.67	0.68
**Social functioning (SF)**	41.4	27.9	52.4	27.1	11.1	26.2	0.40	0.42
**Role emotional (RE)**	43.8	31.0	51.0	29.5	7.2	28.8	0.23	0.25
**Mental health (MH)**	48.4	21.0	57.5	21.2	9.1	18.9	0.43	0.48
**MPI**								
**Pain severity (PS)**	24.3	15.1	34.1	18.7	9.7	16.7	0.64	0.58
**Interference (INT)**	28.4	15.7	37.8	19.4	9.4	14.4	0.60	0.65
**Negative mood (NM)**	41.4	22.4	52.5	24.0	11.1	24.7	0.50	0.45
**Life control (LIF)**	51.4	21.0	57.1	20.9	5.7	23.0	0.27	0.25
**Social and away from home activities (SOC)**	40.8	19.9	44.7	17.8	3.9	14.1	0.20	0.28
**SCL-90-R**								
**Somatization (SOM)**	64.7	16.2	69.7	17.5	5.0	12.0	0.31	0.41
**Anxiety (ANX)**	73.7	22.8	77.3	22.0	3.6	14.0	0.16	0.25
**Depression (DEP)**	61.6	22.5	69.9	22.2	8.3	15.1	0.37	0.55
**Anger-hostility (AH)**	78.5	17.9	83.5	17.1	4.9	14.5	0.28	0.34
**ODI total score**	55.5	12.4	58.5	13.6	3.0	9.9	0.25	0.31
**Back Performance Scale (BPS)**	51.7	26.6	62.6	25.5	10.9	17.7	0.41	0.61
**6 Minute Walking Distance (6MWD; m)**	403.6	155.2	444.4	155.1	40.8	99.2	0.26	0.41

Legend: *SF-36* Short Form 36, *MPI* Multidimensional Pain Inventory, *SCL-90-R* Symptom Checklist-90-Revised, *ODI* Oswestry Disability Index, *SD* standard deviation, *ES* Effect size, *SRM* Standard response mean, *m* meter. 0 = maximum pain/symptoms/disability, 100 = no pain/symptoms/full function

After treatment (follow-up), all scores were higher, indicating improvement and better health. The highest scores were again on the SCL-90-R, the top score being on Anger-hostility (m = 83.5, sd = 17.1). Score differences (follow-up – baseline) were greatest on SF-36 Vitality (mean difference = 12.5, sd = 18.5), SF-36 Social functioning and MPI Negative mood (11.1 each, sd = 26.2 and 24.7 respectively), BPS (10.9, sd = 17.7), and SF-36 Bodily pain (10.2, sd = 13.9). All other score differences ranged between 3.0 (sd = 9.99) (ODI) and 9.9 (sd = 20.6) (SF-36 Role physical). The 6MWD scores between baseline and follow-up differed on average by 40.8 m (sd = 99.2). SF-36 Bodily pain showed the highest ES and SRM (both 0.73). Second highest were SF-36 Vitality, with an ES of 0.67 and an SRM of 0.68. The smallest changes were observed on SCL-90-R Anxiety, where the ES of 0.16 was not significantly different from 0. All other ESs and SRMs reached statistical significance.

### Cross-sectional construct validity

At baseline (Table 3), an excellent association and strong correlation (r ≥ 0.75) was observed between SF-36 Mental health and MPI Negative mood (0.76), and between SCL-90-R Anxiety and Depression (0.79). Of the total of 190 correlations 41 (=21.6%) were moderate to good (0.75–0.50). SF-36 Bodily pain correlated with MPI Pain severity (0.66) and with MPI Interference (0.59). The PBMs correlated with the SF-36 Physical functioning with r = 0.52 BPS, with r = 0.63 6MWD, and with r = 0.56 ODI. A correlation of r = 0.65 was found between the BPS and the 6MWD, the strongest correlation for both PBM. All other correlations between the self-assessments and the BPS or the 6MWD were lower. The ODI correlated highest with MPI Interference (0.66). All other correlations (146/190 = 76.8%) were fair or showed little or no relationship (< 0.50).

**Table 3:** Cross-sectional construct validity: bivariate Pearson correlations of baseline scores

	SF-36								MPI					SCL-90-R				ODI	BPS	6MWD
	PF	RP	BP	GH	VT	SF	RE	MH	PS	INT	NM	LIF	SOC	SOM	ANX	DEP	AH			
**SF-36**																				
**PF**	1.00	0.39	0.36	0.30	0.24	0.35	0.39	0.30	0.31	0.45	0.30	0.19	0.23	0.31	0.24	0.29	0.19	0.56	0.52	0.63
**RP**		1.00	0.45	0.20	0.34	0.40	0.34	0.28	0.37	0.49	0.31	0.14	0.12	0.13	0.11	0.25	0.24	0.40	0.21	0.27
**BP**			1.00	0.25	0.39	0.44	0.28	0.31	0.66	0.59	0.38	0.23	0.27	0.24	0.17	0.31	0.24	0.49	0.28	0.43
**GH**				1.00	0.50	0.38	0.34	0.48	0.30	0.39	0.42	0.36	0.36	0.43	0.47	0.54	0.39	0.38	0.25	0.24
**VT**					1.00	0.52	0.41	0.67	0.38	0.58	0.60	0.52	0.42	0.47	0.51	0.67	0.49	0.44	0.16	0.22
**SF**						1.00	0.52	0.60	0.48	0.72	0.63	0.43	0.46	0.37	0.43	0.58	0.45	0.51	0.18	0.28
**RE**							1.00	0.64	0.25	0.51	0.57	0.32	0.24	0.33	0.42	0.54	0.42	0.37	0.21	0.28
**MH**								1.00	0.32	0.60	0.76	0.52	0.33	0.50	0.67	0.74	0.63	0.41	0.22	0.19
**MPI**																				
**PS**									1.00	0.63	0.41	0.22	0.24	0.25	0.17	0.29	0.25	0.53	0.25	0.32
**INT**										1.00	0.65	0.38	0.41	0.35	0.40	0.54	0.50	0.66	0.32	0.39
**NM**											1.00	0.63	0.42	0.35	0.55	0.66	0.64	0.41	0.13	0.18
**LIF**												1.00	0.36	0.25	0.42	0.53	0.46	0.25	−0.01	0.06
**SOC**													1.00	0.16	0.21	0.39	0.22	0.38	0.07	0.16
**SCL-90-R**																				
**SOM**														1.00	0.71	0.67	0.47	0.43	0.29	0.29
**ANX**															1.00	0.79	0.68	0.35	0.23	0.12
**DEP**																1.00	0.71	0.45	0.18	0.18
**AH**																	1.00	0.31	0.07	0.05
**ODI** total score																		1.00	0.48	0.45
**BPS**																			1.00	0.65
**6MWD**																				1.00

Legend: *SF-36* Short Form 36, *MPI* Multidimensional Pain Inventory, *ODI* Oswestry Disability Index, *SCL-90-R* Symptom Checklist-90-Revised, *BPS* Back Performance Scale, *6MWD* 6 Minute Walking Distance, *PF* Physical functioning, *RP* Role physical, *BP* Bodily pain, *GH* General health, *VT* Vitality, *SF* Social functioning, *RE* Role emotional, *MH* Mental health, *PS* Pain severity, *INT* Interference, *NM* Negative mood, *LIF* Life control, *SOC* Social and away from home activities, *SOM* Somatization, *ANX* Anxiety, *DEP* Depression, *AH* Anger-hostility

At follow-up (Table 4), the following excellent associations were found: SF-36 Mental health with MPI Negative mood (0.76), SF-36 Vitality with Negative mood (0.75), MPI Pain severity with Interference (0.75), SCL-90-R Somatization with Depression (0.77), SCL-90-R Anxiety with Depression (0.84) and with Anger-hostility (0.78), and SCL-90-R Depression with Anger-hostility (0.76). In 62/190 (=32.6%) correlations the associations were moderate to good. SF-36 Bodily pain correlated highest with the ODI (0.66). The PBMs correlated with SF-36 Physical functioning with r = 0.53 BPS, with r = 0.55 6MWD, and with r = 0.69 ODI. A correlation of r = 0.61 was found between the BPS and the 6MWD, which was also the strongest correlation for both PBMs in the follow-up measurements. The ODI correlated highest with MPI Pain severity (0.73). All other correlations (121/190 = 63.7%) were fair or showed little or no relationship (< 0.50).

**Table 4:** Cross-sectional construct validity: bivariate Pearson correlations of follow-up scores

	SF-36								MPI					SCL-90-R				ODI	BPS	6MWD
	PF	RP	BP	GH	VT	SF	RE	MH	PS	INT	NM	LIF	SOC	SOM	ANX	DEP	AH			
**SF-36**																				
**PF**	1.00	0.60	0.56	0.44	0.41	0.28	0.39	0.28	0.54	0.60	0.33	0.29	0.38	0.41	0.32	0.39	0.27	0.69	0.53	0.55
**RP**		1.00	0.58	0.34	0.36	0.25	0.53	0.27	0.53	0.51	0.31	0.24	0.23	0.34	0.21	0.29	0.26	0.58	0.46	0.42
**BP**			1.00	0.34	0.45	0.42	0.45	0.43	0.62	0.60	0.43	0.38	0.46	0.39	0.30	0.41	0.28	0.66	0.39	0.36
**GH**				1.00	0.57	0.34	0.38	0.56	0.54	0.55	0.54	0.46	0.38	0.53	0.58	0.58	0.49	0.53	0.33	0.27
**VT**					1.00	0.52	0.49	0.75	0.49	0.56	0.62	0.60	0.45	0.63	0.55	0.68	0.47	0.49	0.34	0.26
**SF**						1.00	0.53	0.61	0.33	0.54	0.51	0.57	0.39	0.50	0.48	0.56	0.39	0.41	0.18	0.16
**RE**							1.00	0.56	0.39	0.51	0.54	0.41	0.26	0.40	0.38	0.48	0.32	0.51	0.31	0.24
**MH**								1.00	0.41	0.52	0.76	0.68	0.37	0.57	0.67	0.73	0.59	0.44	0.25	0.17
**MPI**																				
**PS**									1.00	0.75	0.46	0.38	0.37	0.53	0.42	0.48	0.38	0.73	0.38	0.37
**INT**										1.00	0.53	0.48	0.53	0.60	0.51	0.60	0.44	0.72	0.34	0.35
**NM**											1.00	0.72	0.34	0.56	0.64	0.69	0.65	0.45	0.29	0.25
**LIF**												1.00	0.37	0.47	0.51	0.59	0.47	0.37	0.16	0.17
**SOC**													1.00	0.37	0.29	0.46	0.27	0.49	0.20	0.26
**SCL-90-R**																				
**SOM**														1.00	0.74	0.77	0.65	0.61	0.32	0.31
**ANX**															1.00	0.84	0.78	0.46	0.27	0.13
**DEP**																1.00	0.76	0.56	0.31	0.25
**AH**																	1.00	0.44	0.20	0.18
**ODI** total score																		1.00	0.49	0.52
**BPS**																			1.00	0.61
**6MWD**																				1.00

Legend: *SF-36* Short Form 36, *MPI* Multidimensional Pain Inventory, *ODI* Oswestry Disability Index, *SCL-90-R* Symptom Checklist-90-Revised, *BPS* Back Performance Scale, *6MWD* 6 Minute Walking Distance, *PF* Physical functioning, *RP* Role physical, *BP* Bodily pain, *GH* General health, *VT* Vitality, *SF* Social functioning, *RE* Role emotional, *MH* Mental health, *PS* Pain severity, *INT* Interference, *NM* Negative mood, *LIF* Life control, *SOC* Social and away from home activities, *SOM* Somatization, *ANX* Anxiety, *DEP* Depression, *AH* Anger-hostility

### Longitudinal construct validity

In the longitudinal construct validity (difference follow-up – baseline score; Table 5), the best correlation of r = 0.67 was between SCL-90-R Anxiety and Depression. Moderate to good correlations were observed in 13/190 (=6.8%) correlations. SF-36 Bodily pain correlated highest with MPI Pain severity (0.55). The PBMs correlated with the SF-36 Physical functioning with r = 0.22 BPS, with r = 0.32 6MWD, and with r = 0.39 ODI. A correlation of r = 0.37 was found between the BPS and the 6MWD, which for both PBMs was the strongest correlation among the longitudinal measurements. The ODI correlated highest with MPI Pain severity (0.50). All other correlations (177/190 = 93.2%) were fair or showed little or no relationship (r < 0.50).

**Table 5:** Longitudinal construct validity: bivariate Pearson correlations of change scores (difference of follow-up – baseline score)

	SF-36								MPI					SCL-90-R				ODI	BPS	6MWD
	PF	RP	BP	GH	VT	SF	RE	MH	PS	INT	NM	LIF	SOC	SOM	ANX	DEP	AH			
**SF-36**																				
**PF**	1.00	0.34	0.40	0.27	0.35	0.18	0.27	0.23	0.36	0.41	0.33	0.27	0.28	0.28	0.11	0.24	0.12	0.39	0.22	0.32
**RP**		1.00	0.34	0.22	0.34	0.20	0.37	0.20	0.44	0.34	0.22	0.23	0.14	0.18	0.10	0.22	0.21	0.34	0.08	0.18
**BP**			1.00	0.29	0.45	0.31	0.34	0.42	0.55	0.46	0.35	0.37	0.24	0.40	0.25	0.33	0.16	0.44	0.22	0.15
**GH**				1.00	0.43	0.24	0.27	0.36	0.32	0.30	0.38	0.39	0.15	0.30	0.35	0.45	0.24	0.18	−0.03	0.17
**VT**					1.00	0.28	0.30	0.60	0.40	0.47	0.51	0.54	0.21	0.31	0.21	0.40	0.28	0.47	0.15	0.22
**SF**						1.00	0.43	0.48	0.15	0.30	0.39	0.44	0.14	0.20	0.33	0.42	0.26	0.19	0.04	0.07
**RE**							1.00	0.46	0.21	0.26	0.40	0.28	0.13	0.18	0.16	0.29	0.17	0.19	0.04	0.13
**MH**								1.00	0.18	0.30	0.60	0.59	0.19	0.29	0.38	0.45	0.36	0.28	0.07	0.14
**MPI**																				
**PS**									1.00	0.65	0.29	0.28	0.19	0.34	0.11	0.23	0.14	0.50	0.16	0.25
**INT**										1.00	0.34	0.29	0.29	0.34	0.25	0.35	0.22	0.47	0.14	0.23
**NM**											1.00	0.68	0.33	0.20	0.32	0.48	0.49	0.30	0.13	0.18
**LIF**												1.00	0.25	0.19	0.24	0.47	0.39	0.28	0.08	0.19
**SOC**													1.00	0.21	0.13	0.26	0.18	0.28	0.07	0.15
**SCL-90-R**																				
**SOM**														1.00	0.61	0.56	0.36	0.38	0.17	0.21
**ANX**															1.00	0.67	0.54	0.20	−0.05	−0.01
**DEP**																1.00	0.61	0.24	0.08	0.14
**AH**																	1.00	0.15	0.03	0.07
**ODI** total score																		1.00	0.20	0.27
**BPS**																			1.00	0.37
**6MWD**																				1.00

Legend: *SF-36* Short Form 36, *MPI* Multidimensional Pain Inventory, *ODI* Oswestry Disability Index, *SCL-90-R* Symptom Checklist-90-Revised, *BPS* Back Performance Scale, *6MWD* 6 Minute Walking Distance, *PF* Physical functioning, *RP* Role physical, *BP* Bodily pain, *GH* General health, *VT* Vitality, *SF* Social functioning, *RE* Role emotional, *MH* Mental health, *PS* Pain severity, *INT* Interference, *NM* Negative mood, *LIF* Life control, *SOC* Social and away from home activities, *SOM* Somatization, *ANX* Anxiety, *DEP* Depression, *AH* Anger-hostility

### Factor analysis

The factor analysis revealed 2 factors for the baseline scores, follow-up scores and score differences (difference follow-up – baseline score) (Table 6). The total explained variance at baseline was 55.3%, at follow-up 60.9%, and for the score differences 44.0%. Overall, a similar pattern emerged in all 3 analyses, with generally weaker factor loads on the score differences.

**Table 6:** Factor loads of baseline scores, follow-up scores and score differences (difference of follow-up – baseline score)

	baseline		follow-up		score differences	
	Psychosocial factor	Pain & Function	Psychosocial factor	Pain & Function	Psychosocial factor	Pain & Function
**SF-36**						
Physical functioning (PF)	0.16	0.73	0.19	0.81	0.18	0.65
Role physical (RP)	0.20	0.56	0.14	0.76	0.19	0.54
Bodily pain (BP)	0.24	0.68	0.31	0.70	0.35	0.62
General health (GH)	0.57	0.27	0.60	0.38	0.53	0.24
Vitality (VT)	0.73	0.27	0.72	0.34	0.50	0.52
Social functioning (SF)	0.63	0.44	0.66	0.21	0.59	0.13
Role emotional (RE)	0.57	0.33	0.48	0.44	0.43	0.30
Mental health (MH)	0.84	0.21	0.86	0.17	0.71	0.22
**MPI**						
Pain severity (PS)	0.27	0.63	0.39	0.68	0.15	0.76
Interference (INT)	0.57	0.63	0.54	0.63	0.31	0.67
Negative mood (NM)	0.80	0.24	0.81	0.22	0.69	0.29
Life control (LIF)	0.68	0.03	0.75	0.15	0.65	0.27
Social and away from home activities (SOC)	0.43	0.25	0.40	0.40	0.25	0.33
**SCL-90-R**						
Somatization (SOM)	0.60	0.23	0.72	0.34	0.51	0.30
Anxiety (ANX)	0.81	0.05	0.84	0.13	0.75	−0.08
Depression (DEP)	0.89	0.16	0.87	0.25	0.82	0.12
Anger-hostility (AH)	0.80	0.04	0.78	0.11	0.71	−0.03
**ODI**	0.36	0.71	0.40	0.78	0.20	0.68
**Back Performance Scale**	0.01	0.69	0.11	0.68	−0.08	0.44
**6 Minute Walking Distance (m)**	0.00	0.79	0.03	0.71	0.01	0.52
**Explained variance (%)**	43.0	12.3	48.9	11.9	33.5	10.5
**Total explained variance (%)**		55.3		60.9		44.0

Legend: *SF-36* Short Form 36, *MPI* Multidimensional Pain Inventory, *SCL-90-R* Symptom Checklist-90-Revised, *ODI* Oswestry Disability Index, *m* meter

In all 3 analyses, the psychosocial factor explained variances between 33.5 and 48.9%. SF-36 Mental health, MPI Negative mood and SCL-90-R Anxiety, Depression and Anger-hostility attained the highest psychosocial factor loads with up to 0.89. In the analysis of score differences, SF-36 Vitality and Role emotional and SCL-90-R Somatization loaded less strongly than at baseline and follow-up.

The pain & function factor explained variances between 10.5 and 12.3%, with the BPS loading moderately (0.69 and 0.68) on this factor at baseline and follow-up, but not on the score differences. The 6MWD loaded 0.79 at baseline and 0.71 at follow-up, but more weakly (0.52) on the score differences. SF-36 Physical functioning and Bodily pain, MPI Pain severity and Interference, and the ODI loaded strongly on the pain & function factor in all 3 analyses. SF-36 Role physical alone loaded strongly (0.76) on the follow-up measurement only.

## Discussion

This study investigated the comprehensive scope of a multidimensional, biopsychosocial approach to the assessment of patients with CLBP, which applied a range of 4 PROMs and 2 PBMs and determined the cross-sectional and longitudinal validity of those measures. The patients were assessed before and after attending a standardized interdisciplinary pain management program for chronic musculoskeletal disorders. Validity was quantified by direct comparison of the scales of the individual validated instruments, as there is no “gold standard” for evaluating PROMs. This is to our knowledge the first study to compare such a large number of generic and disease-specific scales, both self-rated and examiner-based, combined in a single set for the assessment of CLBP.

In our study most bivariate correlations between the scales, both cross-sectional and longitudinal, PROMs and PBMs, were moderate to fair, indicating that those measures reflect somewhat different aspects of disability. The evidence of a relationship between disability, whether self-reported or performance-based, and psychological factors is inconclusive. Independently of the measurement scales used, most correlations and associations between the two as reported in the literature were weak to moderate, and some studies showed no association at all [14, 35, 36]. Moderate correlations were found between PROMs and PBMs for physical function [51], while for work-related limitations large differences between the constructs of PROMs and PBMs were shown [52].

The construct of pain itself is covered by the MPI Pain severity scale alone. Its 3 items focus exclusively on pain, namely the severity and level of pain and the amount of suffering caused by pain [13]. The functional consequences of pain, however, are comprehensively covered by 10 items in the MPI Interference due to pain scale. The SF-36 Bodily pain scale has two items, one quantifying the strength of pain, the other the interference of pain in the performance of activities of daily living. These 3 scales showed moderate correlations, between 0.55 and 0.66 (7/9 correlations of the 3 correlation analyses, with 2 exceptions). The ESs ranged between 0.60 and 0.73 and were among the highest observed effects of all scales. Thus both the chronic-pain-specific MPI and the generic SF-36 performed well in this construct and showed moderate construct overlap. In one longitudinal study after treatment in a multidisciplinary pain center, the same scales reached ESs between 0.41 and 0.44; an overlap between SF-36 Bodily pain and MPI Pain severity was reported with a cross-sectional correlation of r = 0.71 [53].

Among the 6 scales that cover the domain of physical functioning (SF-36 Physical functioning, SF-36 Role physical, MPI Interference, ODI, BPS and 6MWD) 24/28 cross-sectional correlations ranging between 0.42 and 0.72 were found. In the longitudinal analysis, the levels were markedly lower, ranging from 0.20–0.47 in 11/14 comparisons. In this group of scales, the SF-36 Role physical and the BPS showed the lowest correlations, meaning that the construct overlap of those 2 functional scales is moderate to weak. Notably, if we take the BPS as the “gold” standard (for criterion validity) for assessing low back pain function (which would be expected from the a priori construct), we find that the construct convergence to the self-assessment scales is rather weak (correlations between 0.08 and 0.53). Overall, correlation and factor analyses showed that the construct of the self-assessment scales is moderately different from that of the BPS and mainly covers ambulation (e.g. 5/10 items of SF-36 Physical functioning). This is corroborated by the higher correlations of the self-assessment scales to the 6MWD than to the BPS. Finally, the construct of physical function could not be separated from that of pain in the factor analysis.

The ODI was designed as a condition-specific PROM for physical disability or function [30, 38]. Nevertheless, 4 out of 10 items address health constructs other than physical functioning, namely pain intensity, social life, traveling and sleep [54, 55]. The ODI is included in the weaker pain & function factor in all 3 factor analyses (Table 6) indicating multidimensionality, although the current evidence about the dimensionality of the ODI is controversial [40, 56].

The ODI correlated highest with MPI Interference but lower to SF-36 Physical functioning, BPS and 6MWD in our data. On the item level, the ODI includes statements of disability / limitation due to pain, pain changes, support needed from other persons or devices (stick or crutches), and quality of movement. The lumping together of diverse concepts blurs the specific function content [57, 58]. This finding is endorsed by a systematic review of 36 back-specific questionnaires whose constructs included psychosocial and physical functions as well as pain and sleep [54].

It is worth noting that the ODI’s cross-sectional correlations were found to be weak (0.30, 0.35, and 0.41) with three physical performance tests measuring impairment in CLBP [59]. Moreover, the correlations between low-back-pain-specific PROMs and the Isernhagen Work Systems Functional Capacity Evaluation (FCE) in patients with CLBP, which were expected to be strong (r > 0.75), turned out to be moderate to weak (r = 0.52 for ODI), including on the item-level [60]. After spinal operations, the reported correlation between the ODI and SF-36 Physical function was r = 0.77, which is comparable to our correlation at follow-up (Table 4) [61]. Our data showed correlations with the functional tests between 0.45 and 0.52 in the cross-sectional analysis and between 0.20 and 0.27 in the longitudinal analysis.

The mental and especially the affective health domain is covered by SF-36 Mental health, MPI Negative mood, SCL-90-R Anxiety and Depression, and partially by SCL-90-R Anger-hostility. The cross-sectional overlap, with correlations ranging from 0.55–0.84 (12 cross-sectional correlations), was good and the longitudinal overlap, ranging from 0.38–0.67 (6 longitudinal correlations), a little lower but still good enough to show moderate construct overlap. The relatively broad construct of mood / affective symptoms is well covered by the above 5 scales. This finding is supported by the factor analyses, where together those 5 scales built a strong psychosocial factor explaining much more variance than the pain & function factor in each analysis. Together with the results of the responsiveness analysis, the validity of those 5 scales was satisfactory. Although SF-36 Vitality and Social functioning and MPI Life control also loaded on the psychosocial factor, they cover partially different constructs. They are closely, but not solely, related to affective health and showed somewhat less construct overlap in the correlation analysis.

We found no study in the literature that had used correlation coefficients to investigate the mental health scales of the SF-36, the MPI or the SCL-90-R in patients with CLBP. Only one cross-sectional comparison (*n* = 152) reported correlations of the Roland Morris Disability Questionnaire (RMDQ) with SCL-90-R Somatization by r = 0.29, with Anxiety by r = 0.19, and with Depression by r = 0.26 [37]. The correlation of the RMDQ with the ODI was r = 0.80 in the German ODI validation study [30]. In our study the correlation levels of the ODI to the 3 SCL-90 R scales (Somatization, Anxiety, and Depression) were higher than those of the RMDQ listed above.

SF-36 Social functioning and MPI Social and away-from-home activities correlated less than their constructs might lead us to expect: namely, cross-sectionally 0.46 and 0.39 and longitudinally 0.14, indicating that these 2 scales converged weakly. In the factor analyses, both loaded moderately on the psychosocial factor with SF-36 Social functioning loading slightly more strongly.

The CLBP syndrome comprises many more dimensions of health and quality of life than just back-related functioning. The combined use of PROMs and PBMs in this study provided comprehensive and complementary information on pain, psychosocial and physical functioning and limitations, and HRQoL in patients with CLBP undergoing standardized multidisciplinary rehabilitation for chronic musculoskeletal pain. The observation that all scales of the comprehensive, generic SF-36 showed much lower levels of health than expected by the population norms in our former study underlines its multidimensionality [19].

The comprehensiveness of the assessment of CLBP is a strength of this study. All the measurement instruments used in the study are in common clinical use in CLBP populations. They are well studied and have good psychometric measurement properties. The PROMs included generic and disease-specific instruments as well as psychosocial measurements. Exploratory factor analysis provided (biopsychosocial) model-directed findings in contrast to pre-determined, hypothesis-directed, confirmatory factor analysis. Furthermore, patients were assessed not only cross-sectionally, at one point in time, but also longitudinally on the basis of published comparative data. A limitation of our study is that the data were collected in a specific patient population with the diagnosis of CLBP in a multidisciplinary pain program, which might reduce the generalizability of the results.

## Conclusions

The selected set of individually validated measurement scales appeared to provide comprehensive coverage and assessment of the complex, multidimensional CLBP syndrome. This is supported by the high levels of explained variance in the factor analyses and by the observation that all scales in the current assessment set revealed improvement after the multimodal pain program. The picture of the CLBP syndrome was dominated by the psychosocial domain, which explained most of the variance. The need to employ a broad spectrum of measurement constructs was supported by the fact that many scales showed only partial convergence within the same domain.

As expected, the pain, pain interference and function scales of the self-assessments showed high construct overlap with each other and with the functional performance tests. Divergence was seen in the BPS and 6MWD to the psychosocial factor. MPI Interference and the ODI, however, loaded also on the psychosocial factor, whereas SF-36 Social functioning and SF-36 Vitality converged also to the pain & function dimension. High specific construct convergence, especially in the psychosocial domain, was observed on the SCL-90 scales (except Somatization) and MPI Life control and to a lesser degree on the other MPI and the SF-36 scales. All SCL-90 scales (except Somatization) diverged strongly from the pain & function dimension.

The comprehensiveness of the measurement, the consistent findings for cross-sectional and longitudinal outcome and the exploratory nature of the (factor-) analysis may be helpful in the planning and design of future studies or in the assessment of clinical routine. For the clinical outcome measurement of multimodal rehabilitation of CLBP, we recommend a minimum set of instruments consisting of the SF-36 Bodily pain, SF-36 Vitality, SF-36 Social functioning, MPI Interference, SCL-90R Anxiety, and SCL-90R Depression. The findings should be confirmed by further research and the sets should be adapted according to specific therapeutic focus and to research aims.

## Data Availability

All data and material are freely available. Please contact the corresponding author for data requests.

## Abbreviations

6MWD: 6 Minute Walking Distance
BPS: Back Performance Scale
CLBP: Chronic Low Back Pain
ES: Effect size
HRQoL: Health-related Quality of Life
m: Mean
max: Maximum
MPI: Multidimensional Pain Inventory
ODI: Oswestry Disability Index
PBM: Performance-Based Measure
PROM: Patient Reported Outcome Measurement
SCL-90-R: Symptom Checklist-90-Revised
sd: Standard deviation
SF-36: Medical Outcomes Study Short Form 36 Health Survey
SRM: Standard response mean
VAPAIN: Validation and Application of a patient-relevant core set of outcome domains to assess multimodal PAIN therapy
ZISP: Zurzach Interdisciplinary Pain Program (Zurzacher Interdisziplinäres Schmerz Programm)
